# In Vitro and In Vivo Efficacy of New Composite for Direct Pulp Capping

**DOI:** 10.1155/2021/8414577

**Published:** 2021-11-25

**Authors:** Julianne Coelho Silva, Tainah Oliveira Rifane, Antônio Ernando Ferreira-Junior, Ana Paula Alves, Richard Miron, Yufeng Zhang, Pierre Basílio Almeida Fechine, Elayne Valério Carvalho, Victor Pinheiro Feitosa

**Affiliations:** ^1^Postgraduate Program in Dentistry, Federal University of Ceará, Fortaleza, Brazil; ^2^Paulo Picanço School of Dentistry, Fortaleza, Brazil; ^3^Department of Periodontology, University of Bern, Bern, Switzerland; ^4^Wuhan University, Wuhan, China; ^5^Department of Organic and Inorganic Chemistry, Federal University of Ceará, Fortaleza, Brazil; ^6^Division of Oral Pathology, School of Dentistry, University Center Unichristus, Fortaleza, Brazil

## Abstract

**Objectives:**

To investigate physicochemical properties, dentin bonding, cytotoxicity, and in vivo pulp response of experimental self-adhesive composites tailored to direct pulp capping.

**Materials and Methods:**

Experimental composites were prepared with beta-tricalcium phosphate and hydroxyapatite nanoparticles adsorbed with simvastatin and glutathione added at 0% (control resin), 1 wt% (Res 1%), and 10 wt% (Res 10%). A commercial light-curable calcium hydroxide (Ca(OH)_2_) (Ultra-Blend Plus) was used as control material. The physicochemical properties investigated were flexural strength and modulus, calcium release, and degree of conversion. Dentin bonding was assessed by the push-out test. Proliferation and cell counting assays were performed to evaluate in vitro cytotoxicity using fluorescence microscopy. In vivo pulp capping was performed on molars of Wistar rats, which were euthanized after 14 days and evaluated by histological analysis.

**Results:**

No statistical difference was observed in flexural strength and cell viability (*p* > 0.05). Res 10% presented higher modulus than control resin and Ca(OH)_2_. Also, Res 10% attained statistically higher degree of conversion when compared to other experimental composites. Ca(OH)_2_ showed higher calcium release after 28 and 45 days of storage, with no statistical difference at 45 days to Res 10%. All experimental composites achieved significantly higher bond strength when compared to Ca(OH)_2_. While no significant difference was observed in the cell proliferation rates, resins at lower concentrations showed higher cell viability. In vivo evaluation of pulp response demonstrated no pulp damage with experimental composites.

**Conclusions:**

The experimental composite investigated in this study achieved adequate physicochemical properties with minor in vivo pulpal inflammation and proved to be a valuable alternative for direct pulp capping.

## 1. Introduction

Dental caries remains a prominent disease affecting much of the world population, especially in countries with low socioeconomic conditions [1, 2]. Deep caries and traumatic injury to the tooth can lead to pulpal exposure [2]. In this regard, pulp therapy is procedure performed with the aim of maintaining pulp vitality [3]. Standard treatments consist of the application of a material directly onto the exposed tissue [4]. These protective materials should ideally consist of bioactive properties to promote pulp cell activity and pulp healing, and the formation of new reparative dentin [3, 5].

Calcium hydroxide-based products have been recommended for the treatment of exposed pulp due to their ability to stimulate dentin formation and antibacterial activity [4, 6]. However, high solubility and fast dissolution are well-known shortcomings of conventional calcium hydroxide materials [3]. Although there are several materials available, neither of them possesses all desirable characteristics. Therefore, direct pulp capping remains challenging for operative dentistry.

In the last decade, the introduction of simvastatin in dentistry has increased [7, 8]. Hydroxymethylglutaryl-coenzyme A reductase inhibitors (e.g., simvastatin) are used as cholesterol-lowering drugs [9]. Previous papers have observed that some statins may increase the mRNA expression of bone morphogenetic protein 2 (BMP-2) in osteoblasts responsible for bone formation as a result [10, 11]. Furthermore, the combination of statin and *α*-tricalcium phosphate (*α*-TCP) promoted odontogenic differentiation in human dental pulp cells. Moreover, it is possible to observe the formation of mineralized nodules, thereby potentially being a suitable biomaterial for pulp capping [8, 12].

Resin composites are materials with optimal physical properties. However, they cannot be applied directly on the pulp tissue due to their harsh pulp irritation, possible necrosis, and potential glutathione depletion [4, 13–17]. Recent studies have shown that some drugs may be used to reduce their cytotoxicity, such as glutathione, which interacts with resin monomers thereby impairing the entrance in pulp cells and increasing cell viability [18, 19]. Although these two drugs (simvastatin and glutathione) possess several dental pulp therapeutic advantageous properties, to date, they have yet to be investigated in combination, or applied in nanocarriers for controlled release in a potential new therapeutic dental biomaterial.

Thus, the goal of the present study was to evaluate selected physicochemical properties, dentin bonding, and cell proliferation and cell viability of human dental pulp (HDP) cells, of an experimental self-adhesive composite resin containing bioactive nanoparticles adsorbed with simvastatin and glutathione. Furthermore, a second objective was to assay the in vivo response of exposed pulps in Wistar rats to these materials. The study hypothesis was that the incorporation of bioactive particles on experimental composite resin interferes directly in the physicochemical properties, cytotoxicity in pulp cells and in vivo response.

## 2. Materials and Methods

### 2.1. Experimental Composites

The monomeric blend of experimental composite resin was composed of 20 wt% Bisphenol-A-glycerolate-dimethacrylate (BisGMA), 68.5 wt% urethane-dimethacrylate (UDMA), and also 10 wt% glycerol-phosphate -dimethacrylate (GPDM), the acidic functional monomer. The photoinitiator system was composed of a combination of camphorquinone+amine, which were 1 wt% ethyl 4-dimethyl-amine benzoate and 0.5 wt% camphorquinone. Then, the experimental composites were produced by mixing filler particles: (i) Res 0%-without bioactive fillers+50 wt% silanated barium glass fillers (0.7 *μ*m mean size, Esstech Inc., Essignton, USA); (ii) Res 1% wt% hydroxyapatite with glutathione+1 wt% hydroxyapatite with simvastatin+1 wt% beta-tricalcium phosphate+47 wt% silanated barium glass; and (iii) Res 10%-10 wt% hydroxyapatite with glutathione+10 wt% hydroxyapatite with simvastatin+10 wt% beta-tricalcium phosphate+20 wt% silanated barium glass. In total, all composites had 50 wt% resin blend and wt% fillers. Light-curable calcium hydroxide (Ca(OH)_2_) (Ultra-Blend Plus, Ultradent, South Jordan, USA) was used as the control material.

### 2.2. Degree of Conversion

The degree of conversion (DC) of the experimental composites was undertaken following a protocol similar to those previously described by Feitosa et al. [20]. Briefly, each material (*n* = 5) was placed in a glass plate and positioned in a Raman microspectrophotometer (Xplora, Horiba, Paris, France) with 532 nm wavelength argon laser and 3.2 mW power. The spectra were assessed before and subsequent to light activation (40 s; 1200 mW/cm^2^, Valo, Ultradent). All spectra were obtained in a range of 1500–1700 cm^−1^. The peak height was determined subsequent to baseline subtraction and normalization process. The residual unreacted aliphatic carbon–carbon double bond content (% C=C) was determined from the ratio of vibrational intensities of aliphatic C=C bonds (peak at 1637 cm^−1^) against an internal standard (aromatic carbon–carbon double bond peak at 1608 cm^1^) before and 40 s after starting photocuring. Degree of conversion was determined by subtracting the C=C% from 100%. The degree of conversion was calculated by means of the formula

(1) $$\begin{array}{c} \text{DC}=\left(1-\text{Rcured}/\text{Runcured}\right)\times 100. \end{array}$$

### 2.3. Push-Out Bond Strength Test

Sixty extracted human premolars were used after approval by the institutional Research Ethics Committee (protocol #1482602). The teeth were cut 1 mm and 2 mm above cementum-enamel junction perpendicular to the longitudinal axis with slow-speed water-cooled diamond saw (Isomet 1000, Buehler, Lake Bluff, USA) to obtain dentin slabs. A central hole was created in each slab by means of a diamond bur (#1013, KG Sorensen) in a high-speed handpiece under running water.

Fifteen dentin slabs (*n* = 15) with approximately 1 mm thickness were used per material. The slabs were positioned onto glass slides with Mylar strips and experimental composites or Ca(OH)_2_ were applied, in the holes, another glass slide was gently pressed on the top of the dentin slab, and the material was light-cured for 40 s. Specimens were then stored in distilled water at 37°C for 24 h.

The push-out test was performed using a universal test machine (EMIC DL 2000, São José dos Pinhais, Brazil), equipped with 500 N load cell at 0.5 mm/min speed. The displacing tip was centralized, and the push-out force was applied until the displacement of the resinous material from the dentin slab. To express the bond strength in megapascal (MPa), the failure load was recorded in Newton (N) and divided by the area (mm^2^) of the material-dentin interface. The calculation of the area was performed using the formula: 2*π*rh, where “*r*” represents the radius of the circular cavity with the material and “*h*” the thickness of the slab. They were measured with a digital caliper with 0.01 mm precision.

### 2.4. Flexural Strength and Elastic Modulus

**Figure 1:**
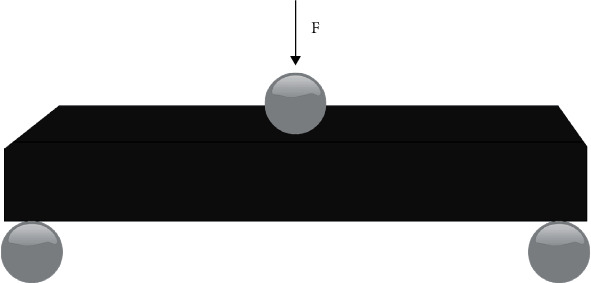
Scheme of flexural strength and elastic modulus evaluation.

Flexural strength and elastic modulus were tested in bar-shaped specimens (25 × 2 × 2 mm, *n* = 10) bar-shaped shown in Figure 1. After 24 h at 37°C and 100% humidity, the specimens were fractured under three-point bending in a universal testing machine (EMIC DL-2000) at 1 mm/min cross-head speed. Flexural strength (FS) and elastic modulus (E) were calculated according to the equations below:

(2) $$\begin{array}{c} \sigma FS=\frac{3\;L\;d}{2\;w\;h^{2}},\\ E=\frac{L.D^{3}}{4\;w\;h^{3}d}, \end{array}$$

where *L* is the failure load (for flexural strength) or the load recorded in the elastic region of the load x displacement curve (for elastic modulus), in Newton, *d* is the distance between the supports (20 mm), *w* is the width and *h* is the thickness of the specimen (both in mm), and *D* is the displacement (in mm) of the cross-head corresponding to *L*. The machine software automatically calculated and reported the maximum flexural strength and elastic modulus in megapascal.

### 2.5. Calcium Release

Six disk-shaped samples (1 mm thickness and 7 mm diameter) from each material (*n* = 6) were prepared using silicone molds, and individually light-cured for 40 s. The samples were stored in 2 mL distilled water, and storage solutions were exchanged after 28 and 45 days with equal volume replacement. The calcium release was determined by mixing the storage solutions with Arsenazo III in 20 mM HEPES at pH 7.4 (Sigma Aldrich, St. Louis, USA). The analysis of calcium release through this solution was performed using a UV-Visible spectrophotometer (Powerwave 340; Biotek, St. Paul, USA) with 656 nm wavelength for 3 s adopting the Arsenazo III colorimetric method [21]. Aliquots of 5 *μ*L of the samples (diluted 1 : 10 and partially neutralized) were added to 50 *μ*L of deionized water before UV-Vis analysis. For calibration, standards containing 40 to 200 *μ*g Ca/mL solutions (Sigma Aldrich) were used.

### 2.6. Cell Culture and Proliferation Assay

Human dental pulp (HDP) fibroblasts and MC3T3 osteoblastic cells (Kargarpour et al. [21]) were seeded onto disks with 6 mm in diameter and 1 mm thick of each material in 96-well plates at a density of 10,000 cells per well either group. During cell seeding, *α*-MEM medium (Invitrogen, Basel, Switzerland) was supplemented with 50 *μ*g/mL ascorbic acid (Invitrogen) and 2 mM *β*-glycerophosphate (Invitrogen) to promote proliferation/differentiation [4]. Cells were quantified using fluorescent MTT assay (Invitrogen) at 1, 3, and 5 days for cell proliferation as previously described ^21^. At desired time points, cells were washed with phosphate-buffered solution (PBS) and quantified using a fluorescence plate reader (Infinite 200, Tecan, Männedorf, Switzerland). Experiments were performed in triplicate with three independent experiments for each condition. Qualitative analysis of resulting cells was obtained by LIVE/DEAD dyes (Sigma Aldrich) using confocal-laser scanning microscopy [4] .

### 2.7. In Vivo Pulpal Response

According to the sample calculation based on the study of Liu et al. [22] that after one week of exposure of the pulp chamber of rats submitted to direct pulp capping treatment, no animals of the control group (0.0%) versus 75.0% of the animals treated with MTA (*n* = 3) presented mineralized tissue deposition, a sample of 6 mice per study group was estimated in order to obtain a sample that represents 80% of the power and 95% confidence to modify the biological behavior after pulp capping with biocompatible materials (Fleiss method with continuity correction). Twelve Wistar rats (*Rattus norvegicus*) were used (*n* = 6) in split-mouth design. Each animal had two of the pulp capping materials randomly applied to the two lower molars according to the material that used: (i) without restorative procedure, (ii) light-curable Ca(OH)_2_, (iii) Res 0%, and (iv) Res 10%. The animals were euthanized by barbiturates (sodium pentobarbital, 150 mg/kg, intraperitoneal) 14 days after the procedure. Hemimandibules were removed surgically and prepared to histological analysis (hematoxylin-eosin) as previously described by Timm 26. Then, qualitative analysis was performed by conventional light microscopy.

### 2.8. Statistical Analysis

All data were compiled and tested by the Shapiro-Wilk normality test and homoscedasticity test (*α* = 0.05). After passing these tests, they were analyzed by one-way ANOVA and Tukey's post hoc test (*p* < 0.05). In the case, variance was not equal, and data were not normal, the Kruskal-Wallis test was performed (*p* < 0.05).

## 3. Results and Discussion

**Figure 2:**
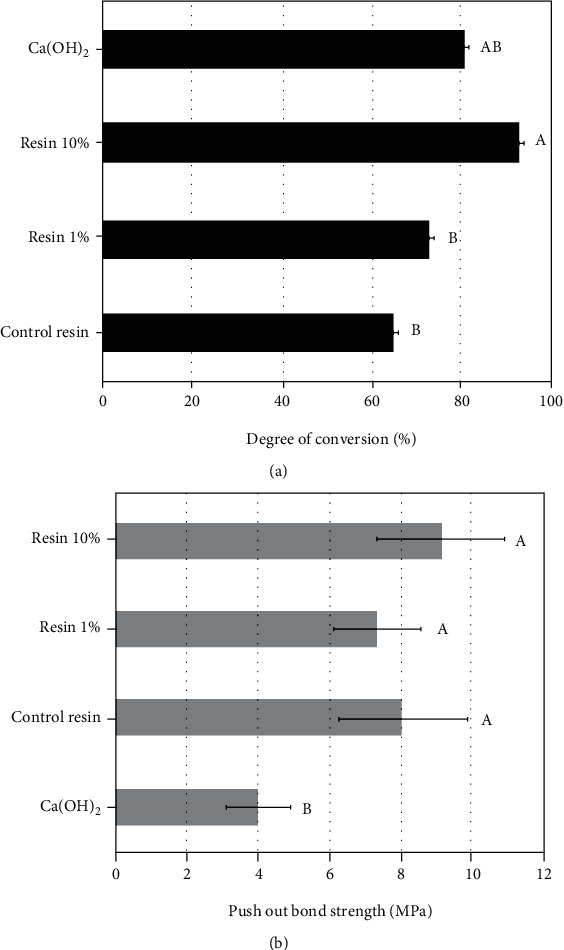
(a) Graph depicting the degree of conversion outcomes. (b) Graph showing the results of push-out bond strength experiment in human dentin. Different capital letters indicate statistically significant differences (*p* < 0.05) among different materials.

**Figure 3:**
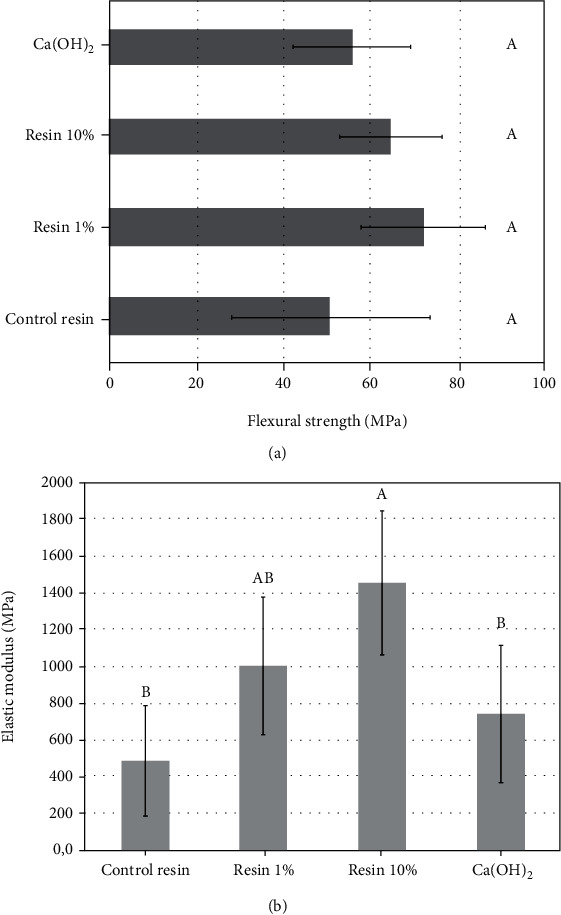
(a) Graph depicting the flexural strength results. (b) Graph highlighting the elastic modulus outcomes. Same capital letters indicate absence of statistically significant differences (*p* > 0.05) among different materials.

**Figure 4:**
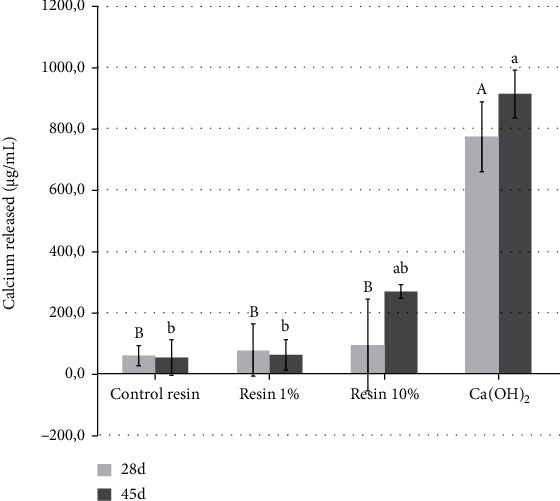
Graph depicting calcium release. Different capital letters indicate statistically significant differences (*p* < 0.05) among different materials during the 28-day period. Different lowercase letters indicate significant differences (*p* < 0.05) between the materials in increase of calcium release from a 28-day period to the analysis of 45 days.

Outcomes of the degree of conversion are depicted in Figure 2(a). No statistical differences were found between groups including Res 0%, Res 1%, and Ca(OH)_2_. Nevertheless, Res 10% attained significantly higher conversion than control resin composite (*p* = 0.004) and Res 1% (*p* = 0.022), but no significant difference was found between Ca(OH)_2_. All experimental composites (Res 0%, Res 1%, and Res 10%) attained push-out bond strength (Figure 2(b)) around 8 MPa, which were statistically higher than Ca(OH)_2_ (mean 4 MPa). The flexural strength (Figure 3(a)) showed an absence of significant differences among all groups (*p* > 0.05). Contrariwise, outcomes of the elastic modulus (Figure 3(b)) demonstrated significant differences among groups. Res 10% exhibited a higher elastic modulus than Res 0% and Ca(OH)_2_ (*p* < 0.001 and *p* = 0.005, respectively), with no statistically significant difference to Res 1% group (*p* = 0.14), which showed intermediate results with no significant difference to all other groups. The findings from the calcium release assay (Figure 4) demonstrated the highest release for Ca(OH)_2_ following 28 and 45 days of storage, without significant difference to Res 10% at 45 days.

**Figure 5:**
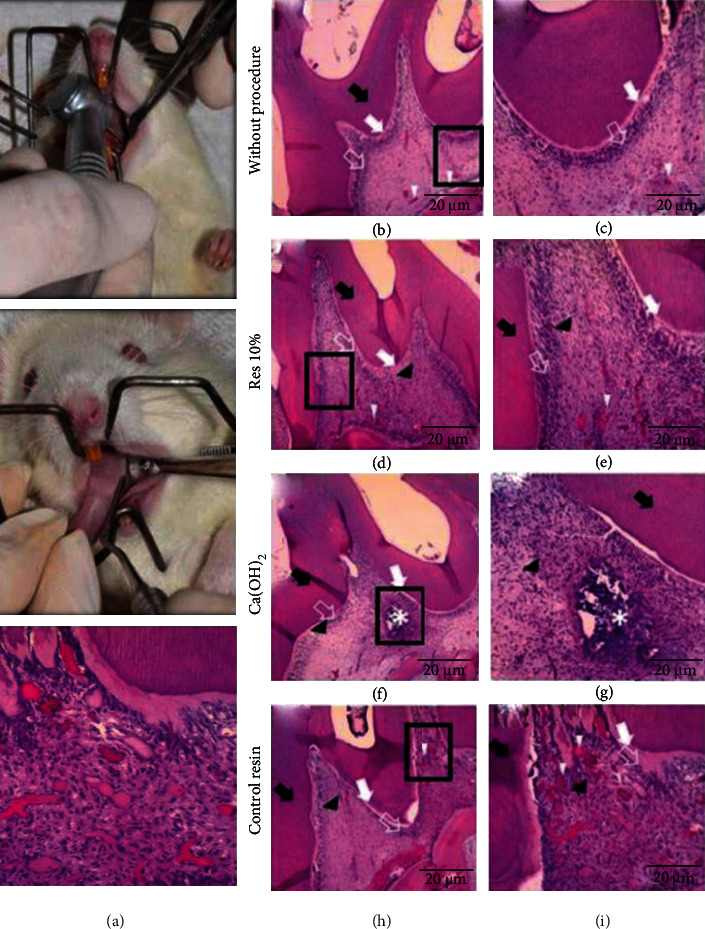
(a) Representative images of in vivo experiment, during the tooth preparation to reach pulp chamber (upper image) and material application (bottom image). (b–i) Hematoxylin-eosin staining of slices obtained from in vivo study of the materials on direct pulp capping. (c), (e), (g), and (i) represent the high magnification (200x) of (b), (d), (f), and (h), respectively. Black arrows indicate dentinal tissue, white arrows show predentin, open white arrows depict odontoblast zone, and black arrows indicate connective tissue and of white arrowheads indicate pulp ectasia (PE). (b, c) A tooth without treatment, in which is possible to observe slight deep PE. (d, e) A tooth treated with Res 10%, which is possible to note moderate PE. (f, g) A tooth restored with Ca(OH)_2_, where necrosis is found and indicated by the white asterisk. (h, i) A tooth restored with control resin (0% bioactive fillers) showing superficial PE and signs of pulp inflammation; higher magnification of this group is found in (i), depicting odontoblast zone and inflammation cells.

The results from the in vivo pulpal response experiment (Figure 5) demonstrated that pulp health and slight cell ectasia were found in the deep pulp in teeth without any procedure. The teeth restored with Res 10% demonstrated moderate cell ectasia in the deep pulp. The findings of the group restored with Res 0% demonstrated large cell ectasia and signs of pulp inflammation. In some teeth restored with Ca(OH)_2_, it was possible to observe cellular damage in a different pattern from previous groups. Furthermore, necrosis areas were present in some teeth treated using Ca(OH)_2_.

**Figure 6:**
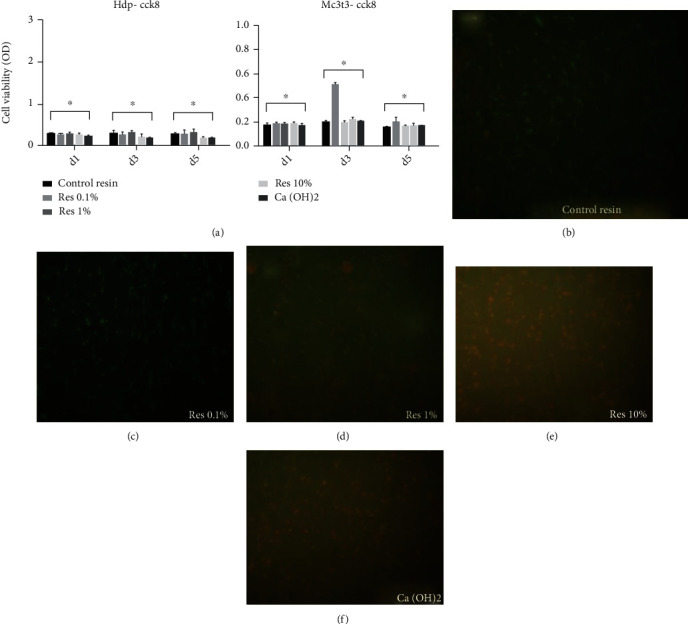
(a) Graph depicting the cell viability results. Asterisk indicates no statistically significant differences (*p* > 0.05) among groups. (b–e) Images obtained by fluorescence microscopy using live/dead dyes for MC3T3 osteoblastic cells submitted to different resins after 5 days storage. (b–e) Cells cultured in control resin, Res 1%, Res 10%, and Ca(OH)_2_, respectively. (b) Relatively intermediary amount of cell death. (c) A smaller amount of cell death when compared to (b). (d) Higher quantity of cell death as the concentration of bioactive particles increased. (e) The increase in the number of dead cells.

The results from the cell proliferation assay (Figure 6(a)) depicted no statistical significant difference in the cell counting among all groups in the culture of fibroblasts. The images from the live/dead assay (Figures 6(b)–6(e)) demonstrated that resins containing bioactive fillers improved the cell viability when compared to the Res 0% group (Figure 6(b)). However, the increase in concentration of the bioactive fillers tended to decrease the rate of cell viability (Figures 6(c) and 6(d)). The least concentration of live cells was observed in the Ca(OH)2 group (Figure 6(e)).

## 4. Discussion

The results herein demonstrated that tested composite resin with high (10%) concentration of nanoparticles is cytotoxic and may cause cellular damage. However, the incorporation of bioactive fillers in an experimental resin in low concentrations reduced its cytotoxicity without significantly impairing their physicochemical properties. Therefore, in the hypothesis that the incorporation of bioactive particles in an experimental composite resin interferes directly in the physicochemical properties, cytotoxicity in pulp cells and in vivo response should be accepted.

According to the findings of degree of conversion (DC), Res 10% demonstrated higher polymerization when compared with resin without nanoparticles (Res 0%). This composite formulation had large amount of drugs (simvastatin and glutathione) adsorbed onto nanoparticles. Both compounds are antioxidants and could have created blocking of free radicals during photoinitiation, thereby hampering polymerization. However, during preparation of these experimental composites, drugs were adsorbed onto hydroxyapatite nanocarriers, rather than dissolved in the comonomer blend. Such a procedure was intentionally undertaken with the aim to avoid polymerization inhibition.

Furthermore, the calcium phosphates (hydroxyapatite and beta-tricalcium phosphate) added to the composite increased polymerization in accordance with Andrade Neto et al. [23] who concluded that crystalline hydroxyapatite nanorods included in resin infiltrants improved the DC. This may occur due to crystallinity of hydroxyapatite, which increases light scattering and consequently the polymerization reaction within the resin matrix [23]. More soluble calcium phosphates, when dissolved in the resin, might also lead to higher polymerization owing to the proton-releasing ability of phosphates, thereby acting as catalysts that may even induce spontaneous polymerization [24–26].

The flexural strength outcomes (Figure 3(a)) depicted no significant difference among groups. However, contrasting elastic moduli were attained (Figure 2(b)), as Res10% showed the highest stiffness. Indeed, a suitable explanation for this finding relies on the higher polymerization of composite resin containing 10% bioactive fillers (Figure 2(a)). Ultra-Blend Plus (Ca(OH)_2_ material) showed lower elastic modulus, which may be attributed to its formulation composed basically of calcium hydroxide with minor concentrations of hydroxyapatite and urethane-dimethacrylate, while the composites presented a great quantity of cross-linking monomers and reinforcing filler particles which improved the rigidity [27, 28].

By analyzing calcium release (Figure 4), light-curable calcium hydroxide (Ultra-Blend Plus, Ultradent) notably demonstrated greater release. In fact, such outcomes were expected thanks to the composition of this material. The high release indeed has occurred because soluble calcium is the base composition of such a material. Furthermore, in accordance with a preliminary study, Ultra-Blend provides the greatest release of calcium ions with a significant difference observed between other cements based on calcium hydroxide, which might also be attributed to the presence of hydroxyapatite [29].

The results from the cell proliferation assay demonstrated no statistical difference among groups (Figure 6), which may indicate that the new pulp capping agents might be a potential replacement for traditional calcium hydroxide in terms of cytotoxicity. However, the images of live/dead obtained by fluorescent microscopy suggested that composite resin with lower concentration (1%) of bioactive fillers (Figure 6(c)) may improve the cell viability when compared with composites containing 10% bioactive fillers and the that without bioactive fillers (0%), which is the conventional composite resin used in dental clinics. These findings may indicate that glutathione in low concentrations is able to reduce the oxidative stress and the cell death caused by resin monomers.

These results corroborate with the findings of Nassar et al. [19] who evaluated the effect of glutathione on cell viability. They concluded that glutathione in controlled concentrations had a protective effect against HEMA cytotoxicity. The high concentration of simvastatin may have decreased cell viability in groups with 1% and 10% bioactive nanoparticles (Figures 6(d) and 6(e)), hypothesis that is supported by the investigation of Asl Aminabadi et al. [7] whose findings revealed a higher rate of pulp inflammation and necrosis by increasing the concentration of simvastatin. Okamoto et al. [12] examined the effects of statin on the cell proliferation of human dental pulp stem cells and demonstrated a suppression of cell proliferation, which was mediated through the inhibition of the mevalonate and Rho pathways caused by statins. Besides, they inhibited actin fiber formation and cell cycle progression that is regulated by Rho [30, 31].

Although a qualitative analysis of the slides produced with rat hemi jaws was presented (Figure 5), it is possible to conclude that calcium hydroxide caused more cellular damage to the pulp. These are in agreement with previous studies, such as that of [32], which found a small area of necrosis in pulps capped with Ultra-Blend Plus. When compared with Res 10%, the standard of damage is very different, likely due to presence of bioactive fillers releasing glutathione and simvastatin, both showing anti-inflammatory properties and control of unfolded inflammatory process [8]. The group without particles demonstrated vastly disorganized pulpal tissue that may indicate necrosis, which was reported in previous studies due to cytotoxicity caused by resin monomers [33].

The findings of this study showed that the incorporation of adsorbed simvastatin and glutathione on calcium phosphate nanoparticles in an experimental resin composite did not impair the physicochemical properties; even increasing the polymerization. However, low concentrations of bioactive fillers improved the cell viability when compared with the traditional resin and light-curable calcium hydroxide. There are preliminary studies which report that simvastatin has been tested and proven both in the laboratory and in vivo the excellent ability to manage pulpal tissue biomodulation. Moreover, glutathione has the ability to reduce the cytotoxicity caused by the resin monomers, and there are no reports so far regarding a composite resin for direct pulp capping that incorporates such bioactive substances.

Indeed, pulp capping is still a clinical challenge in dental medicine where biomaterials still show a relevant annual failure rate [34]. Thus, the major advantage of this composite is their self-adhesive and self-etching properties (Figure 2(b)), eliminating acid conditioning and adhesive application, which when performed directly on the pulp tissue may cause cellular damage and intense inflammation [35–37].

## 5. Conclusions

The clinical relevance of the topic may be the possibility of turning pulp capping procedures faster and more efficient without the use of temporary cements and restorations. Further studies are therefore needed to prove the regenerative properties of pulp tissue and its mineralization over time from this biomaterial.

## Data Availability

Experimental data available upon request.
